# Congenital Heart Disease in East Africa

**DOI:** 10.3389/fped.2019.00250

**Published:** 2019-06-26

**Authors:** Salim G. M. Jivanji, Sulaiman Lubega, Bhupi Reel, Shakeel A. Qureshi

**Affiliations:** ^1^Department of Congenital Heart Disease, Evelina London Children's Hospital, Guy's and St Thomas NHS Foundation Trust, London, United Kingdom; ^2^Uganda Heart Institute, Mulago Hospital Complex, Kampala, Uganda; ^3^Department of Paediatrics and Child Health, College of Health Sciences, University of Nairobi, Nairobi, Kenya

**Keywords:** congenital heart disease, cardiac missions, charity, pediatric cardiology, cardiac surgery, charity, cardiac catheterization

## Abstract

Congenital Heart Disease (CHD) is an enormous problem in Low Middle Income Countries and particularly in sub-Saharan Africa. There is an estimated 500,000 children born in Africa with CHD each year with a major proportion of this in sub-Saharan Africa. The vast majority of these children receive sub-optimal or no care at all. In East Africa: Kenya, Tanzania, and Uganda have all attempted to create a CHD service for the last 20 years with minimal success due to various factors. Visiting cardiac missions have made considerable contributions in the development of CHD services in these countries, however there remains a significant number of children with lack of care. We explore the positive aspects of the current projects, the various factors that hinder growth in this area, and what can be done to promote CHD service growth in these countries.

The incidence of congenital heart disease (CHD) ranges from 19 to 75 per 1,000 live births (1). It is also the leading cause of birth defects and the second leading cause of death in the first year of life after infectious diseases (2).

Factors contributing to birth defects include single gene and chromosomal disorders, environmental teratogens, multifactorial inheritance, and micro-nutrient deficiencies (1, 3). Whilst exposure to medicines and recreational drugs—including alcohol and tobacco—may not greatly contribute to the incidence in low-middle income countries (LMICS) and particularly in sub-Saharan Africa, maternal infectious diseases such as syphilis and rubella greatly increases it (3). Additionally due to the high fertility rate and high neonatal mortality rate, the incidence of CHD in sub-Saharan Africa is greatly underestimated (4).

The burden of CHD is immense. Advancing technology has improved the outcomes of children with these defects in developed countries. However, the financial impact of a child with CHD has exponentially increased over the last few decades. A recent study found that of the total pediatric hospitalizations in the United States, only 3.6% accounted for treating children with CHD. However, it represented more than 15% of the annual costs for pediatric hospitalizations (5). As such in sub-Saharan Africa, the cost burden is significant and carries important implications in treating children with CHD.

## Congenital Heart Disease in Sub-Saharan Africa

The Global Burden of Disease Study estimated that 80% of deaths from non-communicable diseases, including CHD, now occur in LMICs (6, 7). Additionally the World Health Organization (WHO) estimates that 1% of live births have CHD accounting for ~1.5 million per year. There have been estimates by WHO to suggest that 90% of these children have suboptimal or no access to care at all with most of these children are concentrated in LMICs, particularly sub-Saharan Africa (8). These numbers however have been mitigated in recent years, by the rapid rise of the Chinese and the Indians' development of their congenital heart service, and their support to LMICs.

### Health Care Considerations

Any established healthcare system goes beyond hospitals, doctors, nurses, and allied health care providers. A successful healthcare system requires a robust infrastructure, which includes government support, good relations with business and industry, and a university program. Importantly, it should include a continuing education program and good governance (9).

Factors that impact healthcare delivery include affordability, access, and awareness (8). All three of these elements must exist for successful healthcare delivery. In CHD, particularly in sub-Saharan Africa, each of these three factors contributes to numerous children being unable to obtain appropriate care.

### Affordability

Without governmental (or humanitarian) support, the treatment of CHD is unaffordable for the vast majority of the population in sub-Saharan Africa. The costs are prohibitive even though the treatment can sometimes be curative or often have a strikingly positive impact on the individual's clinical status. The current setup, particularly in East Africa, is self-funding for the vast majority of patients with CHD. However, there are many discouraging factors in the pathway. For example, once seen by the cardiologists, numerous tests may be required to confirm the diagnosis and identify the type of intervention. This can include electrocardiogram, echocardiogram, or diagnostic cardiac catheterization, which often have a significant individual cost. As such the economic odds heavily favor the wealthy similar to other developing countries (10). Those who cannot afford the treatment will either accept their fate or attempt to raise some money, often with the help of their families or local village communities. However, the money raised frequently falls short or is too late. In the rare instance where they are able to pay for treatment, the financial impact to the family and their village is considerable, not only in their existing state of affairs, but also for their future planning.

Public hospitals (where the care is free or for a nominal cost) attempt to bridge the cost of the care, but are congested and overwhelmed. Political interference, self-preservation, enhanced private pay for skilled professionals, and lack of funding often impedes these centers, which have made recurrent unsuccessful attempts to start and establish a robust program since the early 1970s (9, 11).

Fortunately in the last decade, there has been progress with the three East African countries (Kenya, Tanzania, and Uganda) all taking steps to address the lack of cardiac services and in some cases making progress to ensure that their citizens have full health coverage. However, there still remains a lot to be done.

### Access

Very few cardiac centers exist in each of these countries (12) and they are always located in the main cities. Some countries, such as Kenya, have adopted the use of helpful but infrequent satellite clinics to provide care for patients, who live outside major cities. However, any treatment, which requires admission into a hospital with cardiac expertise, would result in a long, and arduous journey (11).

### Awareness

Late presentation of CHD in western countries is an exception rather than the rule. This is following numerous educational programs, a wide infrastructure of well-trained medical workforce, and several access points for patients to seek help (13). This infrastructure is an investment that has taken decades to develop. In sub-Saharan Africa, where the setup and educational awareness programmes are not as extensive, providing care for patients with CHD becomes a substantial challenge. Several patients, who have initial benign heart conditions, end up developing secondary irreversible damage, as they are not picked up in their early life. Those with more complex conditions, such as duct-dependent lesions, rarely survive.

## East Africa

### Kenya

Kenya's population is just under 50 million with more than 46% living below the poverty line (14). The under-5 mortality rate is 45 per 1,000, a factor of more than 10 compared with developed countries (Table 1). Encouragingly, the mortality rate has halved in the last 15 years with improvement in public health, with better basic hygiene, wider availability of clean water, and rising uptake of childhood vaccination. The first open-heart surgery, closure of an atrial septal defect, was performed in 1973 (11).

**Table 1:** Comparison of country demographics and congenital heart services provided by each country.

	**Population (millions)^**a**^^1^**	**Infant mortality rate^**b**^^2^**	**Under 5 mortality rate^**b**^^2^**	**CHD surgery**	**Surgical units**	**Catheter interventions**	**Intervention centers**
Kenya	48.5	33.6	45.6	~120	6^*^	~120	6^#^
Uganda	41.5	35.4	49	70^@^	1	90^@^	1
Tanzania	55.5	38.3	54	~40^%^	1		1
UK	65.6	3.7	4.3	>5,500^3^	16^&^	>3,500	20^∧^
USA^*$*^	325.7	5.7	6.6	~39,800^4^	>119^5^	>20,000^6^	166 ^4^
Sweden	9.9	2.3	2.8	~600	2^7^	~300	3^8^
Japan	127	1.9	2.6	>9,000 (15, 16)	121 (15)	>4,800 (17)	100 (16)

a Data Gathered from the World Bank—last update 2016.

b *Child Mortality rate—(Children under 5) expressed as deaths per 1,000 in 2017 (Median)—UNICEF. Infant Mortality Rate (Children under 1 year) expressed as deaths per 1,000 in 2017 (Median)*.

* *Centers, which can perform cardiac surgery in Nairobi with operating theaters, bypass machines (not all centers perform regular procedures). Staff are usually free lance, with local surgeons going from one hospital to another*.

# *All are based in Nairobi (as of 2017). CHD surgical and interventional numbers are annual average between 2015 and 2017*.

@ *CHD surgical and interventional numbers are annual average between 2015 and 2017. Note this number has reported to have increased significantly*.

% *CHD Surgical numbers are annual average between 2015 and 2017. Note this number represents half the total number (only CHD) of annual cardiac surgeries*.

& *as of 2017—UK has 11 pediatric cardiology heart units and 5 adult congenital surgical centers*.

∧ *There are an additional 4 centers, which performs simple adult congenital interventions such as ASD closures*.

$ *For the year 2018*.

Based on the population and WHO report, ~5,000 children require congenital heart surgery in Kenya each year. The number of children who do not receive these interventions is daunting. On an annual basis, Kenya performs between 120 and 150 congenital open-heart operations, with a similar number of congenital catheter interventions (personal communication—local pediatric cardiologists, current cardiology trainees; free-lance anesthetists, intensive care nurses, perfusionists; local consumable suppliers, administrative staff in various Kenyan hospitals, 2019). Approximately 50–100 additional patients receive treatment outside Kenya, most of whom are self-funded. This does not include those with rheumatic heart disease, which accounts for a significant workload in Kenya and sub-Saharan Africa. This aligns to WHO's estimate that the vast majority of CHD patients receive suboptimal or no care at all. The number of procedures performed by the local team has remained desperately low for several years despite no shortage of patients.

Most cardiac operations and catheter procedures take place in Nairobi with only recent commencement of another small programme in Mombasa. There are 6 hospitals capable of performing open-heart operations and cardiac catheterizations in Kenya, of which 4 are private. However, not all of them perform congenital heart interventions. The previously active congenital cardiac programme in Kenyatta National Hospital, a public hospital, has been defunct for over 2 years, with no indication of when it is likely to restart. The Mater Misericordiae Hospital has been running a cardiac programme for the last 20 years through various charitable fundraising activities. Charitable missions, ranging from 3 to 4 annual visits, perform a significant proportion of interventions, with various levels of local involvement. The other private hospitals perform *ad-hoc* congenital procedures. Recently MP Shah Hospital has hosted charitable missions with an ambitious view to establish itself as a fully functioning congenital heart service in Kenya. By using the visiting expertise, they are establishing an infrastructure, slowly aligning the staff training, and ensuring the right skills are recruited, to help establish their goal.

The enhanced implementation of the National Health Insurance Fund (NHIF) in Kenya has improved the affordability and subsidized the care of patients presenting to hospitals (18). Founded in the 1960s, the NHIF is a government state corporation, with a directive to provide health insurance to Kenyans over the age of 18 and their families. The mandate for NHIF is to provide accessible, affordable, sustainable, and quality health insurance for all Kenyan citizens. The fund, as of 2017, had more than 6.3 million members covering more than 25 million of the population, which include dependents of the principal member. There has been a recent impetus by the government to cover a larger population addressing all the three points listed above under “health care considerations.” Since 2016 congenital cardiac procedures are covered under this programme. Although the entire cost is not covered, it provides a significant contribution. The challenge for NHIF coverage is that a considerable proportion of the population is informally employed. This means that mandatory contribution to the NHIF, as those by formally employed individuals, does not take place, leaving them vulnerable to the high cost of health care. However, all Kenyans are eligible for coverage with membership contributions aligned to their income, with the aim for universal health coverage by 2030 (18).

### Tanzania

Tanzania has a population of over 55 million, with approximately half the population below the poverty line (19). The infant and under-5 mortality rate is comparable to the other two East African countries (see Table 1). Like its counterparts, it is going through a renaissance with strengthening economy, resulting in improved health service delivery.

Similar to Kenya the first cardiac surgery dates back to the 1970s. It is not until early 2000, that there was a legitimate investment in bringing about a cardiac service in Tanzania. A task force set up by the Ministry of Health visited countries with established cardiac units, with a view to setting up its first cardiac unit in Tanzania (20).

The first unit opened in 2008 as part of the Muhimbili National Hospital. Visiting missions, particularly from China and neighboring Zimbabwe, heavily supported the initial operations in Tanzania. In its inaugural year alone, more than 100 patients were operated, of which 35% consisted of CHD supported by Chinese medical staff.

In 2013 a new cardiac center, The Jakaya Kikwete Cardiac Institute was opened with funding from the Chinese government designed to run independently of other services within the main hospital. The center has a fully functioning operating theater and a cardiac catheter laboratory. In the 3 years leading up to 2016, a total of 259 cardiac surgeries have taken place with a peak in 2015, when 164 cardiac operations were performed due to government drive inviting overseas cardiac missions to operate in Tanzania. The local team independently operated on a small number of patients a year but allowed them to establish themselves with the help of the visiting team. Half of the cardiac operations consisted of CHD patients, whilst the rest consisted of rheumatic heart disease and other cardiac conditions. Recently the number of independent (by the local team) and mission supported heart surgeries has increased with reports that they are undergoing a robust process in the establishment of an organized CHD service.

### Uganda

Uganda is the smallest of the three East African nations with a population of just over 41 million people. With a similar public health profile compared with its neighboring countries, Uganda is also committed to setting up a CHD service. The first closed heart operation was in 1997 (21). However it has taken some time before the Uganda Heart Institute (UHI) in Kampala was set up in 2012. UHI is slowly building up its cardiac service with the help of visiting charity missions. It is the only center in Uganda, which has a combined open heart and cardiac catheterization service. There are two more institutes, which perform open-heart surgery in small numbers, however very few CHD procedures are performed outside Kampala.

Similar to Tanzania, the focus in Uganda has been to try and establish a CHD service by training and recruiting doctors and surgeons in one center. This process is slowly establishing a reliable service over the last 6 years. Notably, the local medical team perform majority of the procedures and the annual numbers are slowly increasing (see Table 1).

Importantly UHI, from an early stage, has accepted pediatric cardiology rotations and fellowships for training. This is an important investment in the development of this specialty with local graduates.

## Challenges of Developing a Cardiac Program in East Africa

Development in East Africa is progressing rapidly. There has been significant progress in the economy, infrastructure, and primary health care with objective data supporting this in all the three East African countries. However, there remain challenges in developing healthcare particularly specialized cardiac healthcare, on a consistent basis. The following points addresses some of the challenges faced:

1. Inconsistent funding from the government.
2. Charitable missions provide a considerable proportion of CHD interventions. The care is often free and equipment is for nominal costs.
3. Huge variation in individual income results in inconsistent charges for procedures to each family.
4. Challenges in recruiting and retaining staff, due to inconsistent number of congenital heart interventions and poor pay.
5. Unreliable supply of consumables to perform cardiac surgery or interventional procedures.
6. Heavy custom charges and distributor's margin of profit for imported consumables and equipment required for surgery or catheter interventions. This often results in the procedures being more expensive than other well-established countries. As a result the possibility of obtaining items in bulk to offset costs has not developed.
7. Irregular number of cardiac interventions resulting in poor relationships being developed with local procurement agents.
8. Lack of investment by industry and individual donators in supporting the services in Kenya.
9. Corruption at various levels, which has hindered development of services.

It is worth emphasizing that almost all the current centers, which are being established in developing countries, have required the help of visiting missions (see Table 2 for current cardiac missions visiting East Africa). This is essential to offset the heavy cost of implementing these techniques and perhaps more importantly, transferring the experience and training from the visiting teams—locally. It is often not possible for the local team of doctors and nurses to visit developed countries to work and gain experience due to difficulties in obtaining registration of medical/nurses. Additionally funding is often an additional insurmountable barrier. Those who have visited as observers have often found the lack of participation in procedures and decision-making overseas a difficult challenge to convert to their own practice.

**Table 2:** List of Cardiac Charity Missions to East Africa—not exhaustive.

**Kenya**	**Tanzania**	**Uganda**
Healing Little Hearts (UK)	Healing Little Hearts (UK)	Chain of Hope (UK)
World Medical Mission (USA)	Save a Child's Heart (Israel)	Rotary International
Medical & Education Aid to Kenya (UK)	Mending Kids (USA)	World Children's Initiative (USA)
Kothandam Sivakumar Cardiac Mission (India)	CardioStart (USA)	Mission Bambini (Italy)
Slovakia Republic Cardiac Mission (Slovakia)	Open Heart International (Australia)	Samaritan's Purse (USA)
		Hwan Sung (Korea)

*The Chinese cardiac input in Tanzania was a governmental arrangement between the two countries and not strictly a charity organization*.

With the exception of Egypt, Morocco, and South Africa, the rest of Africa is still in the process of trying to provide consistent congenital cardiac care than is currently available. Even these three countries have benefited from regular charity mission visits in the last few decades before establishing their services.

## Discussion

Establishing a CHD service is difficult and has numerous obstacles. The investment (both financially and as an infrastructure) required is significant and must be considered a long-term and progressive project. All three East African countries have been a part of this process for more than 20 years with limited but encouraging results.

For any healthcare project to succeed, it is vital that all three elements: awareness, access, and affordability, need to be addressed. Moreover, in addition to governmental support, a successful healthcare system requires good relations with business and industry (logistical and financial) and a university program to allow continuing educational development.

The development of a successful CHD service should be limited to a few centers. This would allow concentration of patients, resources, expertise, and international assistance. Once sustainability is achieved, these centers can become hubs for the development of even more centers.

All three countries have high quality university medical programmes. Pediatric cardiology needs to be formally recognized as a specialty with rotations in this field, similar to the achievements in Uganda. Hospitals, which do not have pediatric cardiology service, should work with others to allow rotations for their students or trainees to lay the foundation of future development of this specialty. Medical students and junior doctors educational programmes should allow participation in visiting charity missions. Education and training is critical in establishing the service and spreading awareness.

Rarely, some of the local medical teams have been fortunate to visit overseas and be part of an established training programme. This core team is particularly permeable to the incremental expertise brought by visiting international teams.

It is worth noting that all three countries rely heavily on charity missions to carry out relatively small number of CHD procedures. This is a consistent finding in numerous developing countries, where establishment of a CHD service is being attempted or has been established (21–23). In East Africa in the short term, this is necessary to promote development and sustainability of the cardiac services, however, to avoid stagnation of progress, this approach should be considered supportive and governmental in addition to societal buy-in is essential.

Countries such as Kenya are investing in the NHIF with the aim of universal health coverage in the next 15 years. The recent addition of coverage for CHD procedures is progressive, however but remains short of the actual cost. The shortfall remains a significant burden to the local population and needs further development.

In Tanzania, the national cardiac center is part of a large hospital with governmental support ensuring that the local team are paid well and motivated to improve their skills. However, the governmental subsidies specifically for cardiac procedures although helpful remain limited. Uganda in a short time has built up its service by focusing on one center. Development of local motivated medical staff by visiting missions, a key factor, has slowly led to independence in simple congenital open heart and cardiac catheterization procedures.

CHD has a significant burden on families and simple conditions if left untreated can lead to a debilitating condition. This not only creates a considerable burden but can also affect the future earning potential of families, which are already financially constrained.

Lastly, it could be argued that CHD forms a minor part of morbidity and mortality in public health compared with malaria, HIV, malnutrition, and other endemic conditions. It may be debated that to have a greater impact, the focus of sub-Saharan Africa and indeed LMICs should be to tackle these conditions. However, it has been shown in several studies that the development of CHD services has an impact on the wider hospital. By way of a “towing” effect, it raises the standard and performance and improves quality of the existing services, such as intensive care units and management of other specialties (8). It also brings about an increase in experience to manage other important cardiac conditions, such as rheumatic heart disease and Chagas disease. Additionally, those successfully treated form the basis of economic stability in their respective families and contribute to the society.

## Conclusion

CHD is a huge problem. Various estimates suggest that there are close to 500,000 children born with CHD in Africa annually (24). India, over the last 2 decades, has shown that providing high quality cardiac care is achievable at a significantly reduced cost. After 4 decades of faltering starts, countries such as Kenya, Tanzania, and Uganda, are poised to make a spirited effort in addressing this huge issue. By creating “CHD hubs” in these countries, there is every chance that the East African nations can emulate countries like India and develop a robust cardiac service. These hubs can then be used to accept patients and trainees, which would not only further develop their own programme but also reduce the cost of sending patients to western countries.

## Author Contributions

All authors made substantial contributions to conception and design, acquisition of data, participated in drafting the article, and revising it critically for important intellectual content. All authors gave final approval of the version to be submitted.

### Conflict of Interest Statement

SJ is a committee member of Healing Little Hearts. SQ is a Medical Board member for Chain of Hope. The remaining authors declare that the research was conducted in the absence of any commercial or financial relationships that could be construed as a potential conflict of interest.

## Footnotes

^1^https://data.worldbank.org/indicator/SP.POP.TOTL?locations=VN-KE

^2^*Estimates Generated by the UN Inter-Agency Group for Child Mortality Estimation* (UN IGME) (2018). Available online at: http://data.unicef.org

^3^*National Institute for Cardiovascular Outcomes Research – NICOR*. Available online at: https://nicor4.nicor.org.uk/CHD/an_paeds.nsf/vwContent/NCHDA%20Report%20Analyses%202014-17?Opendocument

^4^*The Society of Thoracic Surgeons*. Available online at: https://publicreporting.sts.org/chsd

^5^*American College of Cardiology*. *ACC Congenital Heart Disease Clinic Directory*. Available online at: https://www.cardiosmart.org/Heart-Conditions/Congenital-Heart-Defects/CHD?w_nav=LN

^6^IMPACT Registry: https://cvquality.acc.org/NCDR-Home/registries/hospital-registries/impact-registry

^7^*SWEDEHEART Swedish Cardiac Surgery Registry*. Available online at: http://www.ucr.uu.se/swedcon/arsrapporter

^8^*National Quality Registry for Congenital Heart Disease (SWEDCON)*. Available online at: http://kvalitetsregister.se/englishpages/findaregistry/registerarkivenglish/nationalqualityregistryforcongenitalheartdiseaseswedcon.2279.html

## References

[1] Hoffman JI Kaplan S. The incidence of congenital heart disease. JACC. (2002) 39:1890–900. 10.1016/S0735-1097(02)01886-7 12084585

[2] Causes of Death Geneva: World Health Organization (2008). Available online at: http://www.who.int/healthinfo/global__burden__disease/cod__2008__sources__methods.pdf

[3] Bruneau BG. The developmental genetics of congenital heart disease. Nature. (2008) 451:943–8. 10.1038/nature06801 18288184

[4] Hoffman JI The global burden of congenital heart disease. Cardiov J Africa. (2013) 24:141–45. 10.5830/CVJA-2013-028 PMC3721933 24217047

[5] Simeone RM Oster ME Cassell CH Armour BS Gray DT Honein MA Pediatric inpatient hospital resource use for congenital heart defects: costs congenital heart defects. Birth Defects Res Part A Clin Mol Teratol. (2014) 100:934–43. 10.1002/bdra.23262 24975483 PMC4422978

[6] Lozano R Naghavi M Foreman K Lim S Shibuya K Aboyans V. Global and regional mortality from 235 causes of death for 20 age groups in 1990 and 2010: a systematic analysis for the Global Burden of Disease Study 2010. Lancet. (2012) 380:2095–128. 10.1016/S0140-6736(12)61728-0 23245604 PMC10790329

[7] Lim SS Vos T Flaxman AD Danaei G Shibua K Adair-Rohani H. A comparative risk assessment of burden of disease and injury attributable to 67 risk factors and risk factor clusters in 21 regions, 1990-2010: a systematic analysis for the Global Burden of Disease Study 2010. Lancet. (2012) 380:2224–60. 10.1016/S0140-6736(12)61766-8 23245609 PMC4156511

[8] Dearani JA Neirotti R Kohnke EJ Sinha KK Cabalka AK Barnes RD. Improving pediatric cardiac surgical care in developing countries: matching resources to needs. Semin Thorac Cardiovasc Surg Pediatr Card Surg Annu. (2010) 13:35–43. 10.1053/j.pcsu.2010.02.001 20307859

[9] Dearani JA Jacobs JP Bolman RM III Swain JD Vricella LA Weins tein S. Humanitarian outreach in cardiothoracic surgery: from setup to sustainability. Ann Thorac Surg. (2016) 102:1004–11. 10.1016/j.athoracsur.2016.03.062 27319988

[10] Kumar RK Tynan MJ. Catheter interventions for congenital heart disease in third world countries. Pediatr Cardiol. (2005) 26:241–9. 10.1007/s00246-005-1005-5 16082576

[11] Yuko-Jowi CA African experiences of humanitarian cardiovascular medicine: a Kenyan perspective. Cardiovasc Diagn Ther. (2012) 2012:2231–9. 10.3978/j.issn.2223-3652.2012.07.04 PMC3839188 24282720

[12] Yacoub MH. Establishing pediatric cardiovascular services in the developing world: a wake-up call. Circulation. (2007) 1161876–8. 10.1161/CIRCULATIONAHA.107.726265 17965402

[13] Sharland G. Fetal cardiac screening and variation in prenatal detection rates of congenital heart disease: why bother with screening at all? Future Cardiol. (2012) 8:189–202. 10.2217/fca.12.15 22413979

[14] The United Nations International Children's Emergency Fund Available online at: https://www.unicef.org/kenya/14776020

[15] Takahashi A Kumamaru H Tomotaki A Matsumura G Fukuchi E Hirata Y. Verification of data accuracy in japan congenital cardiovascular surgery database including its postprocedural complication reports. World J Pediatr Cong Heart Surg. (2018) 9:150–6. 10.1177/2150135117745871 29544411

[16] Committee for Scientific Affairs, The Japanese Association for Thoracic Surgery, Masuda M Endo S Natsugoe S Shimizu H. thoracic and cardiovascular surgery in japan during 2015: annual report by the Japanese association for thoracic surgery. Gen Thorac Cardiovasc Surg. (2018) 66:581–615. 10.1007/s11748-018-0968-0 30076512

[17] Pediatric Interventional Cardiology Establishment of the Japanese Society of Pediatric Interventional Cardiology (JPIC) database. Pediatr Cardiol Card Surg. (2015) 31:30–8. 10.9794/jspccs.31.30

[18] Okungu V Chuma J McIntyre D. The cost of free health care for all Kenyans: assessing the financial sustainability of contributory and non-contributory financing mechanisms. Int J Equity Health. (2017) 16:39. 10.1186/s12939-017-0535-9 28241826 PMC5327514

[19] World Bank Tanzania Mainland Poverty Assessment: A New Picture of Growth for Tanzania Emerges. World Bank (2015).

[20] Nyawawa ETM Ussiri EV Chillo P Waane T Lugazia E Mpoki U Cardiac surgery: one year experience of cardiac surgery at muhimbili national hospital, Dar es Salaam, Tanzania. East Centr Afr J Surg. (2010) 15:111–8.

[21] Aliku TO Lubega S Lwabi P Oketcho M Omagino JO Mwambu T. Outcome of patients undergoing open heart surgery at the uganda heart institute, mulago hospital complex. Afr Health Sci. (2015) 14:946. 10.4314/ahs.v14i4.25 25834506 PMC4370076

[22] Noedir A Stolf G Congenital heart surgery in a developing country: a few men for a great challenge. Circulation. (2007) 116:1874–5. 10.1161/CIRCULATIONAHA.107.738021 17965401

[23] Giamberti A Butera G Mvondo CMVE Cirri S Varrica A Moussaidi N. The shisong cardiac center in cameroon: an example of a long-term collaboration / cooperation toward autonomy. Front Paediatr. (2018) 6:188. 10.3389/fped.2018.00188 30018948 PMC6038727

[24] Hewitson J Zilla PPT Children's heart disease in sub-saharan africa: challenging the burden of disease. SA Heart. (2017) 7:18–29. 10.24170/7-1-1964

